# tRNA-Derived Fragments in Podocytes with Adriamycin-Induced Injury Reveal the Potential Mechanism of Idiopathic Nephrotic Syndrome

**DOI:** 10.1155/2020/7826763

**Published:** 2020-06-22

**Authors:** Shanwen Li, Yiwen Liu, Xiaowei He, Xiagang Luo, Huimin Shi, Gaoting Qu, Xianli Wen, Weihua Gan, Jun Wang, Aiqing Zhang

**Affiliations:** ^1^Department of Pediatrics, The Second Affiliated Hospital of Nanjing Medical University, 262 Zhongshan North Road, Nanjing, Jiangsu 210003, China; ^2^Department of Pediatrics, The Affiliated Hospital of Xuzhou Medical University, 99 West Huaihai Road Xuzhou, Jiangsu 221002, China; ^3^Emergency Department, The Affiliated Nanjing Hospital of Nanjing Medical University, 68 Changle Road, Nanjing, Jiangsu 210003, China; ^4^Department of General Surgery, The Second Affiliated Hospital of Nanjing Medical University, 262 Zhongshan North Road, Nanjing, Jiangsu 210003, China

## Abstract

Idiopathic nephrotic syndrome (INS) is a disease involving injury to podocytes in the glomerular filtration barrier, and its specific causes have not been elucidated. Transfer RNA-derived fragments (tRFs), products of precise tRNA cleavage, have been indicated to play critical roles in various diseases. Currently, there is no relevant research on the role of tRFs in INS. This study intends to explore the changes in and importance of tRFs during podocyte injury in vitro and to further analyze the potential mechanism of INS. Differentially expressed tRFs in the adriamycin-treated group were identified by high-throughput sequencing and further verified by quantitative RT-PCR. In total, 203 tRFs with significant differential expression were identified, namely, 102 upregulated tRFs and 101 downregulated tRFs (*q* < 0.05, ∣log2FC | ≥2). In particular, AS-tDR-008924, AS-tDR-011690, tDR-003634, AS-tDR-013354, tDR-011031, AS-tDR-001008, and AS-tDR-007319 were predicted to be involved in podocyte injury by targeting the Gpr, Wnt, Rac1, and other genes. Furthermore, gene ontology analysis showed that these differential tRFs were strongly associated with podocyte injury processes such as protein binding, cell adhesion, synapses, the actin cytoskeleton, and insulin-activate receptor activity. KEGG pathway analysis predicted that they participated in the PI3K-Akt signaling pathway, Wnt signaling pathway, and Ras signaling pathway. It was reported that these pathways contribute to podocyte injury. In conclusion, our study revealed that changes in the expression levels of tRFs might be involved in INS. Seven of the differentially expressed tRFs might play important roles in the process of podocyte injury and are worthy of further study.

## 1. Introduction

Idiopathic nephrotic syndrome (INS) is a glomerular disease that predominantly occurs in children and is characterized by massive proteinuria, hypoalbuminemia, hyperlipidemia, and edema [1, 2]. Podocytes, as an important part of the glomerular filtration barrier, participate in preventing proteins from escaping to Bowman's space [3, 4]. Currently, the viewpoint that podocyte injury is the basic pathology of INS has become well established. However, the exact pathogenesis of podocyte injury has not been elucidated [1, 5]. Therefore, it is of great importance to explore the mechanism of podocyte injury in idiopathic nephrotic syndrome.

Small noncoding RNAs (sncRNAs) are members of the noncoding RNA family and have been found to play crucial roles under many pathological conditions [6]. Recently, a novel class of sncRNAs, transfer RNA-derived fragments (tRFs), obtained by multiple cleavage of tRNAs, has been found to have diverse functions [7, 8]. Our previous research showed that tRFs may regulate the differentiation of podocytes and the process of chronic kidney disease [9]. In addition, a recent study showed that plasma exosomal tRFs might be diagnostic biomarkers for osteoporosis [10]. Furthermore, serum tRFs have been found to serve as potential candidate biomarkers for the diagnosis of nontriple negative breast cancer [11]. However, there have been no relevant reports on the relationship between tRFs and INS in a podocyte injury model.

To explore the potential function of tRFs in podocyte injury, we used adriamycin to establish a model of experimental nephrotic syndrome in vitro [12]. The differential expression profiles of tRFs between the adriamycin-treated group (Adr group) and the normal cell group (NC group) were examined by high-throughput sequencing. The reliability of the tRF sequencing data was verified by quantitative RT-PCR (qRT-PCR). The miRanda algorithm and TargetScan miRNA prediction database were used to predict the target genes of tRFs. Furthermore, gene ontology (GO) and KEGG pathway analyses were performed to predict the potential functions of differentially expressed tRFs. This study attempts to explore the underlying mechanism of podocyte injury from the perspective of tRFs and reveal the potential role of tRFs in the development of INS.

## 2. Materials and Methods

### 2.1. Cell Lines and Culture Conditions

The immortalized mouse podocyte cell line was a gift from Dr. Mundel (Boston, MA, USA) [13]. Cells were grown under growth-permissive conditions to produce a large number of cells. The growth medium consisted of RPMI 1640 medium (Gibco, Gaithersburg, MD, USA) containing 10% fetal bovine serum, 1% penicillin-streptomycin solution, and 1‰ interferon-*γ*. Cells were incubated at 33°C under 5% CO_2_ [14, 15]. To induce differentiation, podocytes were cultured for 14 days in a culture dish coated with type I collagen using a medium containing no interferon-*γ* (nonpermissive conditions), and differentiation was confirmed by assessment of podocyte differentiation markers. Then, differentiated cells were cultured in serum-free RPMI-1640 medium (GIBCO BRL) for 24 hours to synchronize all cells into a quiescent state. Then, the Adr group was treated with adriamycin (1 *μ*g/ml) for 24 hours. Each treatment was repeated three times.

### 2.2. High-Throughput Sequencing

Total RNA samples were pretreated to remove some RNA modifications that interfere with the construction of the RNA-seq library. Then, the 3′ and 5′ miRNA adapters were successively ligated to each sample. cDNA was then synthesized and amplified using Illumina's proprietary RT primers and amplification primers. Subsequently, a PCR-amplified fragment of approximately 135-160 bp was excised from the PAGE gel and purified. Finally, a quantitative analysis of completed libraries was performed using an Agilent 2100 Bioanalyzer. For sequencing using the Illumina NextSeq 500 system, the library samples were processed, denatured, and diluted to a loading volume of 1.3 ml and a loading concentration of 1.8 pM. A NextSeq 500/550 V2 kit (fc-404-2005, Illumina) was used to load the diluted libraries and advance them for sequencing. The libraries were denatured and diluted to a loading volume of 1.3 ml and a loading concentration of 1.8 pM. The diluted libraries were loaded in a NextSeq 500/550 V2 Kit (fc-404-2005, Illumina) and sent to the Illumina NextSeq 500 system for sequencing according to the manufacturer's instructions. The original step in Illumina NextSeq 500 sequencing involves reading the data through the Illumina filter for subsequent analysis. The pruned reading segments (5′ and 3′ adapter bases removed) were compared with mature and pre-tRNA reference sequences. After statistical analysis, valid sequences were retained for subsequent tRF expression profiling and differential expression analysis. The remaining reads were aligned with transcriptome data including miRNA biotypes. Hierarchical clustering was performed and volcano plots were generated in *R* or Python for calculations and graphical analysis of the differentially expressed tRFs. The differentially expressed tRFs are listed in Table 1.

### 2.3. Western Blot Analysis

Total protein was extracted with RIPA lysis buffer (Sigma), and protein concentrations were determined by a BCA assay. Proteins were separated on a 10% SDS-polyacrylamide gel and transferred to a nitrocellulose membrane. The membrane was blocked with a solution of 5% powdered nonfat milk for 2 hours at room temperature. After blocking, the membrane was placed in a solution containing the primary antibody and incubated overnight at 4°C. Then, the membrane was washed and incubated with a 1 : 5000 dilution of the secondary antibody (Sigma) for 2 hours. After washing the membrane again, the enhanced chemiluminescence reagents were used to react with the horseradish peroxidase-conjugated secondary antibody to detect antibody binding, and band densities were quantified using ImageJ (NIH, https://imagej.nih.gov/ij/).

### 2.4. RNA Extraction and qRT-PCR

Total RNA from the NC and Adr groups was prepared for qRT-PCR. Total RNA was extracted using TRIzol reagent (Life Technologies, USA) according to the manufacturer's protocol. To improve the efficiency of qRT-PCR, rtStar™ tRF and tiRNA pretreatment kits (Arraystar, USA) were used to remove the terminal and internal methyl groups from total RNA isolated from podocytes of the NC and Adr groups. The RNA concentration and purity were quantified for each sample by a NanoDrop ND-1000 instrument. Then, following the manufacturer's instructions, a Bulge-Loop™ miRNA qRT-PCR Starter Kit (RiboBio, Guangzhou, China) was used to reverse transcribe RNA to cDNA and carry out PCR amplification. The primer sequences targeting tRFs are listed in Supplementary Table (available here).

### 2.5. Target Gene Prediction

GtRNAdb-based readings were generated using the tRNA database Genomic tRNA Database (http://gtrnadb.ucsc.edu/). Target genes of tRFs were predicted by the miRanda algorithm and TargetScan miRNA prediction database (http://mirdb.org/). The target genes of the top 20 tRFs with the highest fold changes are listed in Table 2.

### 2.6. GO and KEGG Pathway Analyses

To validate the potential functions of differentially expressed tRFs, target genes of differentially expressed tRFs (*q* < 0.05, ∣log2FC | ≥2) were selected and entered into the DAVID (https://david.ncifcrf.gov/tools.jsp) website for GO and KEGG pathway analysis.

### 2.7. Statistical Analysis

One-way ANOVA was employed for statistical comparisons. Statistical analyses were conducted with GraphPad Prism 8 software. Statistical significance was determined from *p* values.

## 3. Results

### 3.1. Experimental Procedure and Establishment of the Podocyte Injury Model

**Figure 1:**
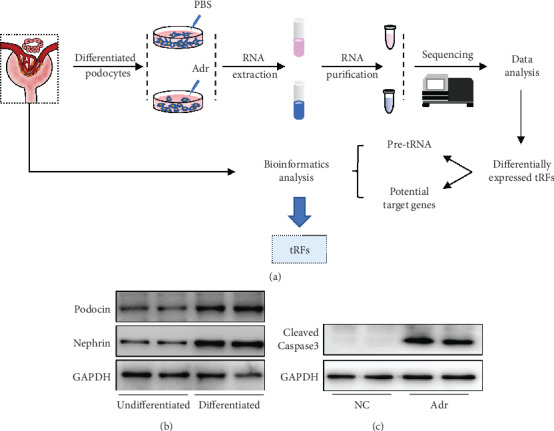
Experimental procedure and establishment of the podocyte injury model. (a) Experimental procedure. (b) Podocyte differentiation. Compared with those in undifferentiated podocytes, nephrin and podocin protein expression levels in differentiated podocytes were significantly upregulated. (c) Podocyte injury. The protein level of cleaved caspase 3 was significantly upregulated in the Adr group.

To investigate podocyte injury-related tRNA-derived fragments, the differentiated podocytes were treated with adriamycin for 24 hours to establish the podocyte injury model. Then, RNA was isolated and purified for high-throughput sequencing. Then, differentially expressed tRFs were selected for variation and bioinformatic analysis (Figure 1(a)). Nephrin and podocin, as slit diaphragm-related molecules, are markers of successful podocyte differentiation [16]. The expression of podocin and nephrin was detected in undifferentiated and differentiated podocytes by Western blot analysis. The results showed that nephrin and podocin were upregulated in differentiated podocytes compared with undifferentiated podocytes, proving successful differentiation of podocytes (Figure 1(b)). Adriamycin is a podocyte toxin that can be used to induce experimental murine nephrotic syndrome [12]. Examination of apoptosis-related indicators in Adr-induced podocytes revealed that the level of cleaved caspase3 was significantly higher than that in the NC group (Figure 1(c)). This result confirmed that the podocyte injury model was successfully established.

### 3.2. Differentially Expressed tRFs between the Normal Cell Group and Adriamycin-Treated Group

**Figure 2:**
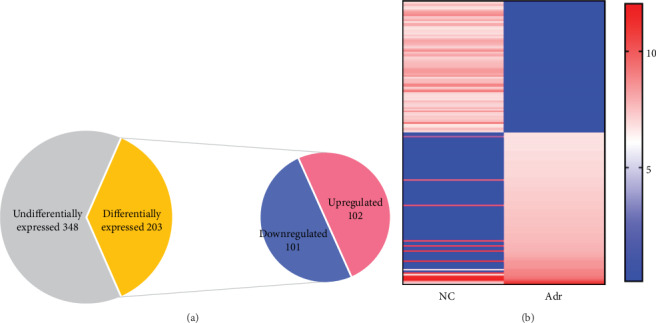
Differentially expressed tRFs in the normal cell group and adriamycin-treated group (a) Number of differentially expressed tRFs. (b) The expression profiles of tRFs show a clear difference between the NC and Adr groups. Red indicates upregulation; blue indicates downregulation; darker shades indicate higher fold changes.

Recent studies have shown that tRFs exist in various diseases, such as infection, inflammation, cancer, and other pathological processes [17]. However, whether these tRFs are related to the pathogenesis of podocyte injury remains to be elucidated. High-throughput sequencing was utilized to detect differentially expressed tRFs between the NC and Adr groups (Table 1) (*q* < 0.05, ∣log2FC | ≥2). The pie charts generated from the results display 551 tRFs, including 102 upregulated tRFs and 101 downregulated tRFs (Figure 2(a)). In addition, the heat maps show the top 20 tRFs with the greatest differences in expression between the NC and Adr groups (Figure 2(b)).

### 3.3. Verification of the High-Throughput Sequencing Results

**Figure 3:**
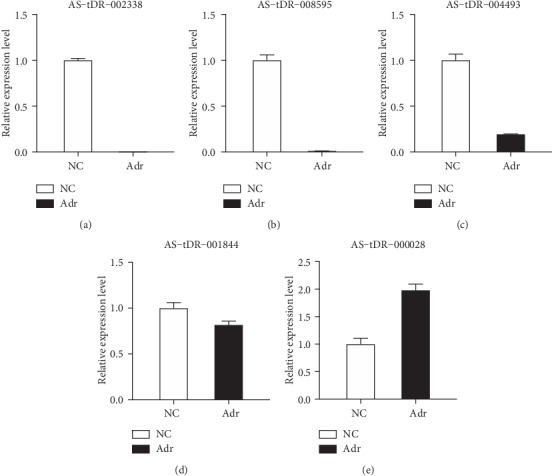
Verification of five randomly selected tRFs Five tRFs with abundant expression were randomly selected for qRT-PCR validation. Among them, AS-tDR-002338, AS-tDR-008595, AS-tDR-004493, and AS-tDR-001844 were downregulated, while AS-tDR-000028 was upregulated (∗*p* < 0.05; ∗∗*p* < 0.01; ∗∗∗*p* < 0.001).

In this study, to prove the reliability of the high-throughput sequencing results, five tRFs (*q* < 0.05) with abundant expression were randomly selected for qRT-PCR validation (Figure 3). The results demonstrated that AS-tDR-002338, AS-tDR-008595, AS-tDR-004493, and AS-tDR-001844 were notably downregulated in the Adr group compared with the NC group. However, AS-tDR-000028 was significantly upregulated in the Adr group compared with the NC group. These results suggested that the high-throughput sequencing results were reliable.

### 3.4. Target Gene Prediction

To date, the regulatory mechanism of tRFs is believed to be highly similar to that of miRNAs. Therefore, to further explore the mechanism of tRFs, target genes of the top 20 differentially expressed tRFs were predicted and are listed in Table 2. Among these tRFs, AS-tDR-008924, AS-tDR-011690, tDR-003634, AS-tDR-013354, tDR-011031, AS-tDR-001008, and AS-tDR-007319 were predicted to be involved in podocyte injury by targeting the Gpr, Wnt, Rac1, and other genes.

### 3.5. GO and KEGG Pathway Analyses of Differentially Expressed tRFs

**Figure 4:**
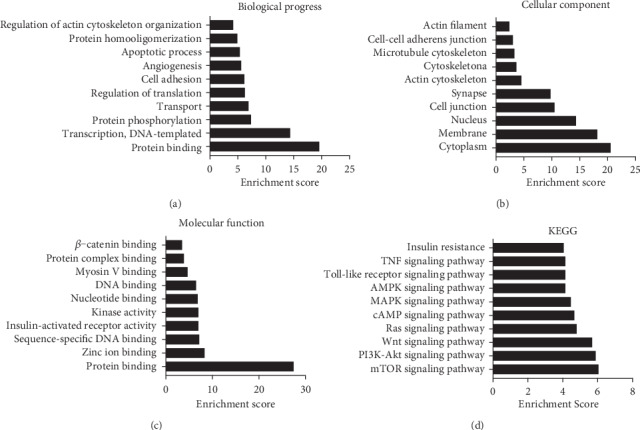
GO and KEGG pathway analyses GO and KEGG pathway analyses of differentially expressed tRFs. (a) Biological process. (b) Cellular component. (c) Molecular function. (d) KEGG pathways.

GO and KEGG pathway analyses were performed to explore the potential functions and mechanisms of these dysregulated tRFs (*q* < 0.05, ∣log2FC | ≥2). GO analysis results consist of biological processes, cellular components, and molecular functions. Enriched categories within these parent categories were obtained. The most highly enriched biological processes were protein binding, DNA template transcription, protein phosphorylation, transport, regulation of translation, cell adhesion, angiogenesis, apoptotic process, protein homooligomerization, and regulation of actin cytoskeleton organization (Figure 4(a)). The most highly enriched cellular components were cytoplasm, membrane, nucleus, cell junction, synapse, actin cytoskeleton, cytoskeleton, microtubule cytoskeleton, cell-cell adherens junction, and actin filament (Figure 4(b)). Sequence-specific DNA binding, protein binding, zinc ion binding, nucleotide binding, insulin-activated receptor activity, kinase activity, DNA binding, myosin V binding, *β*-catenin binding, and protein complex binding were the most highly enriched molecular functions (Figure 4(c)). In addition, KEGG pathway enrichment analysis showed that the mTOR signaling pathway, PI3K-Akt signaling pathway, Ras signaling pathway, Wnt signaling pathway, cAMP signaling pathway, MAPK signaling pathway, AMPK signaling pathway, Toll-like receptor signaling pathway, TNF signaling pathway, and insulin resistance pathway were highly enriched in the differentially expressed tRFs (Figure 4(d)).

## 4. Discussion

Idiopathic nephrotic syndrome with proteinuria is predominantly related to podocyte disease. Podocytes serve as components of the glomerular filtration barrier and play an integral role in preventing urinary protein loss [18]. While the endothelium and glomerular basement membrane can establish protein filtration barriers in a manner dependent on size-selective mechanisms, comprehensive studies have revealed that podocyte injury plays a fundamental role in the pathogenesis of proteinuria [19, 20]. However, compared with other resident cells of the glomerulus, such as mesangial and endothelial cells, podocytes are relatively incapable of efficiently proliferating and replenishing damaged cells after injury.

Commonly, as adaptor molecules, tRNAs function primarily to carry nucleotides for the translation of mRNA and synthetic polypeptide chains [21]. Recent studies have shown that through precise cleavage of both mature and precursor tRNAs, tRFs with diverse functions can be produced [7]. It has been reported that tRFs can be grouped into no fewer than three types, including the 5′-tRF (tRF-5), 3′-tRF (tRF-3), and tRF-1 series. In addition, internal tRFs (i-tRFs), typically derived from the internal region of mature tRNAs, are another novel class of tRFs.

Along with the advancement of genome sequencing technology, tRFs have been proven to participate in the occurrence of various diseases. For instance, tRF-1001 from the Ser-TGA tRNA precursor transcript is highly expressed in a wide range of cancer cell lines. The knockdown of tRF-1001 can impair cell proliferation and suppress DNA synthesis [22]. In addition, it has been shown that the expression level of mature tRNA^Glu^-derived tRF3E is decreased in the blood of epidermal growth factor receptor-positive patients. Through competitive interaction with nucleolin, tRF3E leads to the release of p53 mRNA, thereby exerting tumor-suppressive effects [23]. However, the mechanism of podocyte injury from the perspective of tRFs has not been reported in INS. In our study, high-throughput sequencing was used to determine the expression profile of tRFs in order to investigate the relationship between tRFs and podocyte injury. According to the sequencing data, 551 tRFs were detected, including 102 upregulated tRFs and 101 downregulated tRFs. The verification results for AS-tDR-002338, AS-tDR-008595, AS-tDR-0044935, AS-tDR-001844, and AS-tDR-000028 indicate that the sequencing results are reliable.

Analysis of tRF target genes revealed that multiple tRFs are closely related to podocyte injury. According to these results, AS-tDR-008924 and AS-tDR-011690 can function by targeting G protein-coupled receptors. Several studies support the idea that podocyte damage may accumulate through persistent G protein-coupled receptor activation [24, 25]. In addition, AS-tDR-003634 and AS-tDR-013354 play regulatory roles by binding to Wnt target genes. Wnt/*β*-catenin signaling activation has been confirmed in adriamycin-induced mouse podocytes and may indicate a novel therapeutic strategy for proteinuric kidney diseases [19, 26]. Further, AS-tDR-011031, AS-tDR-001008, and AS-tDR-007319 function by combining with Rac1. Furthermore, AS-tDR-011031, AS-tDR-001008, and AS-tDR-007319 function by interacting with Rac1. Previous studies have shown a causal relationship between excessive Rac1 activity and podocyte injury in a dose-dependent manner [27]. These studies demonstrated that tRFs may play a crucial role in the mechanism of podocyte injury.

GO analysis was used to investigate the potential functions of tRFs. In the biological process category, protein binding and cell adhesion, which are important for podocyte function, had high enrichment scores. As a key element of the GFB, podocytes are permanently exposed to transcapillary filtration pressure. Based on this physiological structure, podocytes must be adhered tightly to the underlying GBM. Integrin *α*3*β*1, *α*2*β*1 and *α*v*β*3, *α*-dystroglycan, syndecan-4, and type XVII collagen are cell-matrix adhesion receptors expressed by podocytes and can tightly connect podocytes to GBM [28, 29]. The cellular components cell junctions, synapses, and actin cytoskeletons had high enrichment scores. Similar to neurons, podocytes are rich in foot processes. These two types of cells share similar molecules for transmembrane transport, signal transduction, and intercellular contact. The adjacent podocyte processes form a characteristic interdigital pattern, which remains between the filtering slits bridged by the glomerular slit diaphragm. Foot processes are further characterized by the presence of highly ordered parallel, contractile actin filament bundles and a podosome-like, cortical network of short branched actin filaments. These actin-based contractile apparatuses are vital factors in kidney function [30, 31]. In the molecular function category, protein binding had the highest enrichment score. In addition, the molecular function insulin-activated receptor activity was enriched. Diabetic nephropathy (DN) is the most common type of end-stage renal disease worldwide, and a significant decrease in the number of podocytes is the strongest predictor of DN. Insulin is an important substance for podocyte survival and plays a critical role in slowing the development of DN [32, 33]. These GO analysis results were consistent with the pathological changes associated with podocyte injury.

KEGG pathway analysis revealed that the PI3K-Akt signaling pathway, Wnt signaling pathway, and Ras signaling pathway had high enrichment scores. A growing number of studies have revealed that the PI3K-Akt signaling pathway is one of the crucial antiapoptotic pathways in podocytes. Dai et al. established an Adr-induced podocyte injury model and found that blocking Akt activation promotes caspase-3 activation in podocytes, indicating that the protective effect against podocyte injury is largely mediated by the PI3K-Akt pathway [34]. Wnts belong to the family of secreted glycoproteins. Interaction of Wnts with the cell membrane receptor Frizzled and the coreceptor LRP5/6 induces a series of downstream signal transduction events, including dephosphorylation of *β*-catenin. While Wnt signaling appears to be silenced in the adult kidney, reactivation of Wnt/*β*-catenin signaling occurs in certain types of kidney diseases, including those with dysfunctional podocyte homeostasis [19, 35]. In addition to the activation of these pathways, activation of the Ras signaling pathway is an essential factor in the pathogenesis of chronic kidney disease [36]. As the principal and active mediator of RAS, angiotensin II can activate TGF-*β*1 and NF-*κ*B signaling to promote renal inflammation and fibrosis. These results reveal that differentially expressed tRFs might play a key role in podocyte injury through various signaling pathways [37, 38] .

## 5. Conclusion

In conclusion, a total of 203 differentially expressed tRFs were identified. Prediction of target genes for these tRFs revealed that seven tRFs, namely, AS-tDR-008924, AS-tDR-011690, tDR-003634, AS-tDR-013354, tDR-011031, AS-tDR-001008, and AS-tDR-007319, play a regulatory role by targeting the Gpr, Wnt, Rac1, and other genes. In addition, the bioinformatic analysis revealed that tRFs could be involved in podocyte injury through the PI3K-Akt signaling pathway, Wnt signaling pathway, and Ras signaling pathway. Our study revealed that changes in the expression levels of tRFs might be involved in INS. Seven of the differentially expressed tRFs might play important roles in the process of podocyte injury and are thus worthy of further study.

## Figures and Tables

**Table 1:** Differently expressed tRFs^∗^.

tRF ID	tRF sequence	Type	Log_2_FC	*q* value
AS-tDR-011816	TCGACTCCGGCTCGAAGGACCA	tRF-3	8.42	<0.001
AS-tDR-016239	GCTTAGTTGGTTAAAGCGCCTGTC	i-tRF	8.38	<0.001
AS-tDR-016240	CTTAGTTGGTTAAAGCGCCTGTC	i-tRF	8.38	<0.001
AS-tDR-008924	TGGTGGGTGCATATGTTTT	tRF-1	8.35	<0.001
AS-tDR-011827	AGCAGCCGGAATTCTATTTT	tRF-1	7.88	<0.001
AS-tDR-011031	GGTAGTGTGGCCGAGCG	tRF-5	7.67	<0.001
AS-tDR-016258	CTTAGTTGGTTAAAGCGCCTGT	i-tRF	7.67	<0.001
AS-tDR-006555	GCCCCAGTGGAACCACCA	tRF-3	7.61	<0.001
AS-tDR-001008	GGCTCGTTGGTCTAGGGGTATGATTCT	tRF-5	7.49	<0.001
AS-tDR-007319	GGCTCGTTGGTCTAGGGGTATGATTCTCA	tRF-5	7.49	<0.001
AS-tDR-012929	GAGTCTACCTAGACTTTTGAGCACAGGATTT	tRF-1	7.49	<0.001
AS-tDR-012944	ACCGCGTGCATTCCTTT	tRF-1	7.49	<0.001
AS-tDR-016265	AGGCAGGCTTTTTCTTT	tRF-1	7.42	<0.001
AS-tDR-007197	GGGGATGTAGCTCAGTGGTAGAGT	tRF-5	7.42	<0.001
AS-tDR-016257	GCTTAGTTGGTTAAAGCGCCTGT	i-tRF	7.42	<0.001
AS-tDR-012914	TGGTGGGTGCATATGTTT	tRF-1	7.35	<0.001
AS-tDR-010656	TCCCTGTGGTCTAGTGGTTAGGATT	tRF-5	7.35	<0.001
AS-tDR-006953	TCGAATCCCAGCGGTGCCTCCA	tRF-3	7.35	<0.001
AS-tDR-011447	GTCACGGTGGCCGAGTGG	tRF-5	7.35	<0.001
AS-tDR-001980	TGGTAGAGCATTTGACT	i-tRF	7.35	<0.001
AS-tDR-012947	CTTCGGATCAGAAGATTGAGGGTT	i-tRF	7.35	<0.001
AS-tDR-008916	GCGTTTGGAAGAGATATTTT	tRF-1	7.28	<0.001
AS-tDR-011894	GTTTATGTGGGAAGTGATATATTATT	tRF-1	7.28	<0.001
AS-tDR-016255	TCGGATCAGAAGATTGAGGGTT	i-tRF	7.28	<0.001
AS-tDR-011983	GTCAGGATGGCCGAGCA	tRF-5	7.20	<0.001
AS-tDR-002346	TGAATCCAGCGATCCGAGTT	i-tRF	7.20	<0.001
AS-tDR-000089	TCGAATCCTGCCGACTACGCCA	tRF-3	7.12	<0.001
AS-tDR-009049	ATCCCACTCCTGACACCG	tRF-3	7.12	<0.001
AS-tDR-012908	CCCACTTCTGACACAGTACTCTTTT	tRF-1	7.12	<0.001
AS-tDR-005979	ATCACGTCGGGGTCACCA	tRF-3	7.12	<0.001
AS-tDR-016244	CTGCGGATCAGAAGATTCTAGGTT	i-tRF	7.12	<0.001
AS-tDR-012941	TTCAGTTTACTCATGGC	tRF-1	7.03	<0.01
AS-tDR-016250	TCAGCCTCCGGAGCTGGGGATTGTGGGT	i-tRF	7.03	<0.01
AS-tDR-016242	TCTGTGTTCTTTATTTC	tRF-1	6.94	<0.01
AS-tDR-011893	GTTTATGTGGGAAGTGATATATT	tRF-1	6.94	<0.01
AS-tDR-005104	TCGTAAACCGAAGATCGCGGGT	i-tRF	6.94	<0.01
AS-tDR-016248	ACAGGCCCATAGTCGAGGGGCACTCTTTC	tRF-1	6.84	<0.01
AS-tDR-006678	GTCCCACCAGAGTCGCCA	tRF-3	6.84	<0.01
AS-tDR-011825	AGGGCTGGTGAGATGGCTC	tRF-1	6.84	<0.01
AS-tDR-008656	CATTTGACTGCAGATCAAGAGGTCCCTGGT	i-tRF	6.84	<0.01
AS-tDR-011466	CACTCTGGACTTTGAATT	i-tRF	6.84	<0.01
AS-tDR-014517	TCGGCTGTTAACCGAAAGGTTGGTGGA	i-tRF	6.84	<0.01
AS-tDR-016259	CTGACTGCGGATCAGAAGATTGTAGGTT	i-tRF	6.84	<0.01
AS-tDR-012938	AGCTGAAGCGTTTTTTT	tRF-1	6.73	<0.01
AS-tDR-001032	GACGAGGTGGCCGAGTGG	tRF-5	6.73	<0.01
AS-tDR-001688	TGGATAGCGCATTGGAC	i-tRF	6.73	<0.01
AS-tDR-001971	AGTGGTAGAGCATTTGACT	i-tRF	6.73	<0.01
AS-tDR-002232	ACTCTGGACTTTGAATC	i-tRF	6.73	<0.01
AS-tDR-009409	TCGGCTGTTAACCGAAAGGTTGGTGGC	i-tRF	6.73	<0.01
AS-tDR-000078	ACCCCACTCCTGGTACCA	tRF-3	6.62	<0.01
AS-tDR-006904	TCCCGGCGGAGTCGCCA	tRF-3	6.62	<0.01
AS-tDR-012688	TGGACATATGTCTGCGTGGGC	i-tRF	6.62	<0.01
AS-tDR-016260	CTGCGGATCAGAAGATTGTAGGTT	i-tRF	6.62	<0.01
AS-tDR-016264	GCTTAGTTGGTTAAAGCGCCTGTT	i-tRF	6.62	<0.01
AS-tDR-001844	CGGGAGACCGGGGTTCGATTCCCCGACGGGGA	i-tRF	3.99	<0.001
AS-tDR-011784	ACACTTGTCAGTTTCTTTT	tRF-1	2.04	<0.001
AS-tDR-008940	TCGACTCCCGGTATGGGAACCA	tRF-3	2.03	<0.001
AS-tDR-000744	GCCGTGATCGTATAGTGGTTAGTACTCTGCG	tRF-5	-1.54	<0.001
AS-tDR-003929	AGTCGGTAGAGCATGAGA	i-tRF	-2.23	<0.001
AS-tDR-007270	TCTCATAATCTGAAGGTCGTG	i-tRF	-2.23	<0.001
AS-tDR-003928	AGTCGGTAGAGCATGAG	i-tRF	-2.29	<0.001
AS-tDR-007329	ACTCTTAATCTCAGGGTCGTG	i-tRF	-2.34	<0.001
AS-tDR-007220	AGTCGGTAGAGCATGAA	i-tRF	-2.40	<0.001
AS-tDR-004493	CCCATAACCCAGAGGTCGATG	i-tRF	-2.46	<0.001
AS-tDR-008595	CTCTTAATCTCAGGGTCGTG	i-tRF	-2.46	<0.001
AS-tDR-002338	TAATGGTTAGCACTCTG	i-tRF	-2.62	<0.001
AS-tDR-006217	CCCCACGTTGGGCGCCA	tRF-3	-6.58	<0.01
AS-tDR-001849	CTGTCACGCGGGAGACC	i-tRF	-6.58	<0.01
AS-tDR-004500	CTCATAATCTGAAGGTCGTG	i-tRF	-6.58	<0.01
AS-tDR-016261	GTGGTCTAGTGGTTAGGATTCA	i-tRF	-6.58	<0.01
AS-tDR-006001	ATCCCACTCCTGACACC	tRF-3	-6.70	<0.01
AS-tDR-007196	GCGTTGGTGGTATAGTGGTGAGCATAGCTA	tRF-5	-6.70	<0.01
AS-tDR-012894	TAGAATTCTCGCCTGCCAT	i-tRF	-6.70	<0.01
AS-tDR-016270	ATTTAGCTCAGCGGTAGAGC	i-tRF	-6.70	<0.01
AS-tDR-000086	TCCGAGTCACGGCACCA	tRF-3	-6.80	<0.01
AS-tDR-006553	GCCCACCCAGGGACGCCA	tRF-3	-6.80	<0.01
AS-tDR-012921	CCCCGGCATCTCCACCT	tRF-1	-6.80	<0.01
AS-tDR-011427	CTGTCACGCGGGAGACT	i-tRF	-6.80	<0.01
AS-tDR-012871	GGTTAGTACTCTGCGTC	i-tRF	-6.80	<0.01
AS-tDR-007302	GCGTTTGTGGTATAGTGGTGAGCATAGCT	tRF-5	-6.90	<0.01
AS-tDR-006242	CCCCGTCCGTGCCTCCA	tRF-3	-6.90	<0.01
AS-tDR-012949	GGTAGAGCATGAGACTT	i-tRF	-6.90	<0.01
AS-tDR-014260	CTGTCACGCGGGAGACCGGG	i-tRF	-6.90	<0.01
AS-tDR-011898	GCCCCGGCATCTCCACCA	tRF-3	-6.99	<0.01
AS-tDR-008363	TTGAGGCTCCAGTCTCTTCGGGGGCGTGG	i-tRF	-6.99	<0.01
AS-tDR-013309	CCCATAACCCAGAGGTCGAT	i-tRF	-6.99	<0.01
AS-tDR-013365	ATTAGCTCAGTTGGGAGAG	i-tRF	-6.99	<0.01
AS-tDR-000112	TCCCGGCCAATGCACCA	tRF-3	-7.08	<0.001
AS-tDR-012948	ATGGATAAGGCATCAGT	i-tRF	-7.08	<0.001
AS-tDR-015866	TGTAGTTCAATGGTAGA	i-tRF	-7.08	<0.001
AS-tDR-003757	CGGTCTAAGGCGCTGGA	i-tRF	-7.24	<0.001
AS-tDR-008265	GTTTCCGTAGTGTAGTA	tRF-5	-7.31	<0.001
AS-tDR-006978	TCGATTCCCGGGCGGCGCACCA	tRF-3	-7.31	<0.001
AS-tDR-008401	ACTCTTAATCCCAGGGTCGTG	i-tRF	-7.38	<0.001
AS-tDR-016235	GTTTCCGTAGTGTAGTGGTCATCACGCTA	tRF-5	-7.45	<0.001
AS-tDR-008525	CTTCAAACCTGTAGCTG	i-tRF	-7.45	<0.001
AS-tDR-009392	TGGTTAGGATTCGGCGCTT	i-tRF	-7.45	<0.001
AS-tDR-002080	GACTCTGAATCCAGCGATCCG	i-tRF	-7.51	<0.001
AS-tDR-009252	CTGATAACGCCAAGGTCGCGGGT	i-tRF	-7.51	<0.001
AS-tDR-012926	TCGATCCCCGGCATCTCCACT	tRF-1	-7.57	<0.001
AS-tDR-010681	TCCCCGGCACCTCCACT	tRF-1	-7.57	<0.001
AS-tDR-007205	CTGACACGCGAAAGGTCCCCGGT	i-tRF	-7.57	<0.001
AS-tDR-008548	ATTCCCATTCTTGCGACCCGG	i-tRF	-7.57	<0.001
AS-tDR-000469	TCCTCGTTAGTATAGTGGTGAGTATCCCCG	tRF-5	-7.63	<0.001
AS-tDR-008569	ACTCTTAATCTCAGGGTCGTA	i-tRF	-7.69	<0.001
AS-tDR-007236	AGCGGTCTAAGGCGCTGGA	i-tRF	-7.74	<0.001
AS-tDR-013219	TAGTGGTTAGGATTCGG	i-tRF	-7.74	<0.001
AS-tDR-008281	TCTTAATCTCAGGGTCGTG	i-tRF	-7.79	<0.001
AS-tDR-007303	GCATGGGTGGTTCAGTGGTAGAATTCTT	tRF-5	-7.85	<0.001
AS-tDR-005384	CACGCGAAAGGTCCCCGGT	i-tRF	-7.85	<0.001
AS-tDR-008600	AATGGATAAGGCATCAGC	i-tRF	-7.85	<0.001
AS-tDR-007230	GCATGGGTGGTTCAGTGGTAGAATTCTCA	tRF-5	-7.89	<0.001
AS-tDR-005279	CTCACACGCGAAAGGTCCCCGGT	i-tRF	-7.94	<0.001
AS-tDR-007229	ACTCCAGATCAGAAGGCTGCGTG	i-tRF	-8.03	<0.001
AS-tDR-008301	TCTCATAATCTGAAGGTCGTA	i-tRF	-8.03	<0.001
AS-tDR-004492	CCATAACCCAGAGGTCGATG	i-tRF	-8.07	<0.001
AS-tDR-008570	ACACGCGAAAGGTCCCCGGT	i-tRF	-8.27	<0.001
AS-tDR-008594	CTCTTAATCTCAGGGTCGTA	i-tRF	-8.27	<0.001
AS-tDR-008586	CTCTTAATCCCAGGGTCGTG	i-tRF	-8.31	<0.001
AS-tDR-003556	ATCGTATAGTGGTTAGTACTCTG	i-tRF	-8.38	<0.001
AS-tDR-013354	CTGTTAACCGAAAGGTTGGTG	i-tRF	-8.57	<0.001
AS-tDR-003634	TAGTGGTTAGTACTCTG	i-tRF	-8.74	<0.001
AS-tDR-010713	GAAGATCGCGGGTTCGA	i-tRF	-8.82	<0.001
AS-tDR-011690	ATGGACATATGTCTGCGTG	i-tRF	-9.29	<0.001

^∗^ *q* < 0.05, ∣ log2FC  | ≥2.

**Table 2:** Target genes of differently expressed tRFs.

tRF ID	Type	Putative targets (target score ≥90)^∗^	Log_2_FC	*q* value
AS-tDR-011816	tRF-3	Afap1l2	8.42	<0.001
AS-tDR-016239	i-tRF	Cntd1; Ptpn20; Tead1; Dennd1b; Bach2; Tmbim6; Lgr4; 1700057G04Rik; Arhgap23; Mycn; Grip1	8.38	<0.001
AS-tDR-016240	i-tRF	Plpp3; Spred1; Fyn; Rnf139; Rbm24; Kcnq5; Lrp1; Tex21; Slc38a2; Lrrc19; Tmem165; Arpp19; Gfap; B3galt2; Lrif1; Rbms1; Asf1a; Ubl3; Zhx3; Sema6d; Kcnb2; Nkrf; Aebp2	8.38	<0.001
AS-tDR-008924	tRF-1	Cd300lb; Phip; Slc6a8; Clec16a; Cd84; Tspear; Cacna2d2; Cx3cl1; Jpt1; 2900026A02Rik; Ywhah; Bloc1s6; Gpr158; Pax8	8.35	<0.001
AS-tDR-011827	tRF-1	Limk1; Ngef	7.88	<0.001
AS-tDR-011031	tRF-5	Egfl6; Ash1l; Rab2a; Gna11; Rictor; Il6st; 4930402H24Rik; Rab23; Gorab; Rab12; Kif5b; Wasl; Fmnl2; Gpatch11; Zeb2; Fmn1; Utrn; Fam208b; Itgav; Ptpn23; Osbp; Akt1s1; Taok1; Kdelr2; Kat2b; Hectd1; Rac1; Acbd5; Bod1; Strn3; Cfl2; Tgfbr1; Mark3; Fyco1; Slc37a3; Tardbp; Fkbp1a; Atf7ip; Inpp5a; Twf1; Smug1; Dirc2; Stam; Tnrc18; Tnks; Fabp4; Suco; Morf4l2; Hgs; Atp2a2; Pawr; Rock2; Tab2; Arntl; Rheb; Tor1aip2	7.67	<0.001
AS-tDR-016258	i-tRF	Plpp3; Spred1; Fyn; Rnf139; Rbm24; Kcnq5; Lrp1; Tex21; Slc38a2; Lrrc19; Tmem165; Arpp19; Gfap; B3galt2; Lrif1; Rbms1; Asf1a; Ubl3; Zhx3; Sema6d; Kcnb2; Nkrf; Aebp2	7.67	<0.001
AS-tDR-006555	tRF-3	Mdga2; Slc2a9; Clec10a; Foxj1; Elavl3; Gstt1; Slc8a1; Neurl1a; Sash3; Ppp3r1; Ptov1; Ubp1; Sirt1; 6720489N17Rik; Nufip2; Ranbp9	7.61	<0.001
AS-tDR-001008	tRF-5	Slain1; Rac1	7.49	<0.001
AS-tDR-007319	tRF-5	Slain1; Rac1	7.49	<0.001
AS-tDR-007229	i-tRF	Aak1; Pgm3; Zfp935; Pcmtd2; Pgf; Krt5; Boc; 6720489N17Rik; Gcnt2; Zfp930; AW209491; Mynn; En1; Fam168a; Tbx4; Zfp960; Zfp97; Slc9c1; Satb1; Fryl; Ntm; Ikbip; Eml6; Zfp951; Ptprb; Psme3; Elmod1; Acvr2b; Map4k2; Fshr; Egln1; Ubn1; Hs3st1; Trmt2b; Phka1	-8.03	<0.001
AS-tDR-004492	i-tRF	Cbx3; Trpv5; Mgea5; Lhx9; Mmp12; Dlgap4; Amfr; Slc18a2	-8.07	<0.001
AS-tDR-008570	i-tRF	Wif1	-8.27	<0.001
AS-tDR-008594	i-tRF	Usp21; Unc79; Tmem56; Btbd1; Mmd; Phip; Prtg; Trim33; Cep44; Plxna2; Pappa; Runx1; Sema3e; Dpp10; Kdm6b; Gmfb; Ptchd3; Mtdh; Ube2e1; Npas3; Ocln; Adcyap1; Rab27b; Stk39; Arl8b; Kdm7a; Hcn1; Fut10; Htra4; Rap2c; Fos; Arl5a; Inpp5a; Steap2; Pcmtd1; Tm9sf1; Fadd; Dner; Bcl10; Cdyl2; Mtm1; Tfam; Cdc7; Lhcgr; Tfap4; Plekhm3; Ifih1; Pde1c; Lamp3; Synpr; Pclo; Rc3h1; Med13; Atxn3; Nefl; Map4k3; Eif4e; Naaladl2; Amd2; Fam126b; 4930430F08Rik; Itga4; Calm1; Mab21l2; Fshb; Usp47; Rgs8; Slc35a3; Tsc22d2; Lrrc36	-8.27	<0.001
AS-tDR-008586	i-tRF	Usp21; Unc79; Tmem56; Btbd1; Mmd; Phip; Prtg; Trim33; Cep44; Plxna2; Pappa; Runx1; Sema3e; Dpp10; Kdm6b; Gmfb; Ptchd3; Mtdh; Ube2e1; Npas3; Ocln; Adcyap1; Rab27b; Stk39; Arl8b; Kdm7a; Hcn1; Fut10; Htra4; Rap2c; Fos; Arl5a; Inpp5a; Steap2; Pcmtd1; Tm9sf1; Fadd; Dner; Bcl10; Cdyl2; Mtm1; Tfam; Cdc7; Lhcgr; Tfap4; Plekhm3; Ifih1; Pde1c; Lamp3; Synpr; Pclo; Rc3h1; Med13; Atxn3; Nefl; Map4k3; Eif4e; Naaladl2; Amd2; Fam126b; 4930430F08Rik; Itga4; Calm1; Mab21l2; Fshb; Usp47; Rgs8; Slc35a3; Tsc22d2; Lrrc36	-8.31	<0.001
AS-tDR-003556	i-tRF	Slc44a5; Nkx2-1	-8.38	<0.001
AS-tDR-013354	i-tRF	Twistnb; Pcna; Ifi44; Tmem170b; Zfp37; Erich5; Rab21; Crot; Rfx3; Gnptg; Zfp616; Serpine2; Fgfr2; Msl3l2; Bicd1; Mtf2; Ank3; Fbxl5; Cdc40; Jarid2; Ppp1r36; Idh3a; Rfwd3; Cav2; Nufip2; Clns1a; Smim8; Tmem100; Sass6; Fmnl2; Akirin1	-8.57	<0.001
AS-tDR-003634	i-tRF	Ifit2; Slc16a6; Rala; Epb41l2; Rnf19a; Fgf9; Bmp2; Pdgfra; Abhd5; Wnt9a; Dnm3; Klf9; Tgfbr1; Hdac7; Tssk2; Dpysl2; Bloc1s2; Ror1; Mpv17; Creb3l1; Katnbl1; Capn1; Mmd; Eif4g2; Zfp800; Ptgr2; Col4a3; Lrp4; Arhgef40; Heg1; Cps1; Kctd12b; Erc2; Car9; Cbll1; Med13; Ms4a10; Lhfpl2; Gm11487; Chac2; Entr1; Psma3; Rbbp4; Igfbp5; Wnt11; Vcpip1; Stradb; Nucks1; Coro2a; Naf1; Egr2; Tmem204; Galnt16; Vps18; Srcap; Hs2st1; Sept2; Strn3; Mlph; Vezf1; Rfx7; Dlgap1	-8.74	<0.001
AS-tDR-010713	i-tRF	1190002N15Rik	-8.82	<0.001
AS-tDR-011690	i-tRF	Adam7; Rlim; Simc1; Rwdd3; Slc35e2; Kat6b; Sptlc1; Irgm1; Brwd1; Gpr22	-9.29	<0.001

^∗^Target score based on microRNA target prediction database.

## Data Availability

The data that support the findings of this study are available from the corresponding author upon reasonable request.

## Abbreviations

INS: Idiopathic nephrotic syndrome
sncRNAs: Small noncoding RNAs
tRFs: Transfer RNA-derived fragments
Adr group: Adriamycin-treated group
NC group: Normal cell group
qRT-PCR: Quantitative RT-PCR
GO: Gene ontology
DN: Diabetic nephropathy.

## Supplementary Materials

Supplementary Materials Supplementary Table: primers for qRT-PCR.
