# Supplementary material for: tRNA-Derived Fragments in Podocytes with Adriamycin-Induced Injury Reveal the Potential Mechanism of Idiopathic Nephrotic Syndrome
Source: Biomed Res Int. 2020 Jun 22;2020:7826763. doi: 10.1155/2020/7826763 (PMC7330628; doi:10.1155/2020/7826763)
Supplement: Supplementary Materials — Supplementary Table: primers for qRT-PCR. [file 7826763.f1.docx]

**Supplementary Table. Primers for** **qRT-PCR**

| **Primers** | **Sequence** |
| --- | --- |
| AS-tDR-002338*-F* | 5’-AGCCCGTAATGGTTAGC-3’ |
| AS-tDR-002338*-R* | 5’-CAGTGCAGGGTCCGAGGT-3’ |
| AS-tDR-008595*-F* | 5’-AGCCCGGTCGTATCCAGTGCAGGGTCC  GAGGTATTCGCACTGGATACGAC-3’ |
| AS-tDR-008595*-R* | 5’-CAGTGCAGGGTCCGAGGT-3’ |
| AS-tDR-004493*-F* | 5’-AGCCCGCCCATAACCCAGAG-3’ |
| AS-tDR-004493*-R* | 5’-CAGTGCAGGGTCCGAGGT-3’ |
| AS-tDR-001844*-F* | 5’-AGCCCGCGGGAGACCGGGGTTCGATTCCCCGA-3’ |
| AS-tDR-001844*-R* | 5’-CAGTGCAGGGTCCGAGGT-3’ |
| AS-tDR-000028*-F* | 5’-AGCCCGTCGAATCCTGCCGACT-3’ |
| AS-tDR-000028*-R* | 5’-CAGTGCAGGGTCCGAGGT-3’ |
